# Validation and quantification of major biomarkers in ‘Mahasudarshan Churna’- an ayurvedic polyherbal formulation through high-performance thin-layer chromatography

**DOI:** 10.1186/s12906-020-02970-z

**Published:** 2020-06-11

**Authors:** Prabhjot Kaur, R. C. Gupta, Abhijit Dey, Tabarak Malik, Devendra Kumar Pandey

**Affiliations:** 1 Department of Biotechnology, Lovely Faculty of Technology and Sciences, Lovely Professional University, Phagwara, Punjab 144411 India; 2 Department of Botany, Punjabi University, Patiala, Punjab 147002 India; 3 Department of Life Sciences, Presidency University, Kolkata, India; 4 Department of Biochemistry, College of Medicine and Health Sciences, University of Gondar, Gondar, Ethiopia

**Keywords:** Mahasudarshan churna, *Swertia chirata*, HPTLC, Standardization, Polyherbal formulations

## Abstract

**Background:**

Mahasudarshan Churna (MC) is a polyherbal Ayurvedic medicine that is employed in fever (especially chronic type), cold and malaria, improvement of digestion and appetite, removes toxins from the blood, boosts immunity and protects against common bacterial infections.

**Methods:**

Validation and quantification of oleanolic acid (OA), ursolic acid (UA), mangiferin (M), gallic acid (GA), quercetin (Q) and curcumin (C) in commercial MC formulations by HPTLC method. Mobile phase, hexane: ethyl acetate: acetone (16.4: 3.6: 0.2, v/v) was used for the separation of OA and UA; ethyl acetate: glacial acetic acid: formic acid: water (20: 2.2: 2.2: 5.2 v/v) for the development of M; and toluene: ethyl acetate: formic acid (13.5: 9: 0.6 v/v) for the separation of GA, Q and C in crude sample extracts. Visualization and scanning were performed at λ = 530 nm for OA and UA, at λ = 254 nm for M and at λ = 366 nm for GA, Q and C. In addition, HPLC-PDA analysis was used to confirm the HPTLC results.

**Results:**

Major bio-active compounds in MC formulations were oleanolic acid (1.54–1.78%), mangiferin (1.38–1.52%) and gallic acid (1.01–1.15%); followed by ursolic acid (0.79–0.98%), curcumin (0.45–0.67%) and quercetin (0.22–0.34%).

**Conclusion:**

Analysis of bio-active compounds in the present study was performed using HPTLC methods and later HPTLC results were compared with HPLC. These two methods give comparable results and there was no statistically significant difference between the mean values for all extracts. Present study concluded that this HPTLC technique is low cost, fast, precise, and accurate which can be employed for the quantification of xanthonoid (M), triterpenoids (OA, UA) and phenolics (GA, Q and C) in samples/formulations. Furthermore, present HPTLC method can be conveniently employed for routine quality control analysis of all the six marker compounds in marketed Ayurvedic/herbal formulations.

## Background

Ayurveda system of medicine is beneficial to mankind, but still there is a lack of proper standardizing techniques for the assessment of their quality, quantity and efficiency. According to WHO guidelines, chromatographic techniques are reliable and effectively implemented to standardize and quantify the major biomarker compounds from single drug and polyherbal formulation. The application of medicinal plants in curing diseases is the oldest prevailing system to deal with illness. In ancient civilized countries like India, China, Egypt, Africa, South America, etc. 80% population depends upon traditional Medicine (TM) system to cure innumerable deadly diseases like cancer, AIDS, Malaria etc. A number of indigenous systems such as Siddha, Ayurveda and Unani medicines reveal the existence of around 800 medicinal plants [1]. In the twentieth century, the development of synthetic medicines has led to a decline in the use of herbal medicines. In recent years, herbal medicines have gradually gained considerable approval and fame due to the extensive research of pharmacological effects in human health care. Although much attention and research has been attempted in current years, there is still insufficient information about the phytochemical and metabolomics of herbal medicines, which has created a major challenge in standardization methods [2]. Herbal drugs or phytomedicine should follow the proper safety and efficacy principles for the well-being of mankind [3]. Hence, framework has been required for the quality control and standardization protocols of the herbal drugs.

Ayurveda formulations are of two types: prepared only from one herb and poly herbal formulations that are prepared from the combination of many herbs [4]. As defined in the literature, maximum quality control of herbal ingredients focuses on observational tests viz. macroscopic, microscopic and physicochemical studies [5]. Therefore, standardization protocols using non-conventional analytical practices are required for authentication of herbal/ poly herbal Ayurvedic formulations. At present, phytochemical screening of bioactive marker compounds becomes more effective in the authentication of herbal drugs, and thus helps to reduce adulteration [6, 7].

Mahasudarshan Churna (MC) is a polyherbal ayurvedic medicine that is extensively used in treatment of all types of fever, cold, malaria, liver and spleen diseases [8]. MC helps to improve digestion and appetite; removes toxins from the blood; boosts immunity and protects against common bacterial infections; promote perspiration and urination. MC formulation is known for antimalarial, antioxidant, antipyretic, antiviral and anti-diabetic properties [9]. MC consists of Kiratatikta (*Swertia chirata)* as a principal component along with other 49 Ingredients [10].

*Swertia chirata* Buch.-Ham. ex C. B. Clarke (Family: Gentianaceae) is a bitter medicinal plant native to temperate regions all over the world. *Swertia* genus is present at the high altitudes of western and eastern Himalayas in India, Nepal and Bhutan regions. This herb is also recognized as kirata Tikta, kairata (Sanskrit), Qasabuzzarirah (Arabic, Farsi), Chiravata (Urdu) and Chiretta (trade name). Whole plant of *Swertia chirata* is also exported from India for medicinal purpose [1]. Traditionally, whole plant of *Swertia chirata* has been used to treat various illnesses such as malaria, cough, cold, chronic fever, tremor fever, liver disorders, stomach disorders, asthma etc. [11]. Among all the compounds extracted from *Swertia* genus, important phytochemical bio-markers are xanthonoids, secoiridoids, triterpenoids [12, 13]. Major xanthonoid compound is mangiferin that shows important pharmacological effects such as anti-oxidant, antidiabetic, antiparkinson, anti-inflammatory, chemopreventive, hypoglycemic, cardiotonic and diuretics properties [14]. Major triterpenoids are Oleanolic acid (OA) and Ursolic acid (UA) that are also considered as significant phytochemical bio-marker compounds [15].

To determine the efficacy and authenticity of herbal drugs, chromatographic based analysis have been acknowledged internationally [2]. High-performance Thin-Layer Chromatography (HPTLC) has been developed as an effective technique for the recognition and quality control of some polyherbal formulations viz. ‘Trikatu Churna’; ‘Draksharishta’; ‘Entoban capsules’; ‘Sitopaladi churna’; ‘Hingavastaka churna’; ‘Avipattikara churna’; ‘Sringyadi churna’; ‘Talisadya churna’ [16–18]. Some physicochemical parameters for the standardization of MC formulation have been investigated [10]. Development of standardization methods of this important formulation based on modern analytical technique is required. The objective of current work was the development and validation of HPTLC technique based on the major bio-markers (xanthonoid-mangiferin, triterpenoids- oleanolic acid and ursolic acid, phenolic compounds-gallic acid, quercetin and curcumin) for proper standardization of MC formulation. This is the first time reporting for the qualitative and quantitative estimation of these significant bio-active compounds in MC formulation.

## Methods

### Plant materials and chemicals

Three commercial brands of ‘Mahasudarshan Churna’ (MC) viz. Dabur (MC1), Baidyanath (MC2) and Zandu (MC3) were procured from store of Ludhiana, Punjab (India). The authentic standards - Mangiferin, Oleanolic acid, Ursolic acid, gallic acid, curcumin and quercetin standards were purchased from Sigma-Aldrich (USA). All chemicals and solvents used in our experimentation were of HPLC grade.

Matured plants of *Swertia chirata* Buch.-Ham. ex C. B. Clarke were collected in flowering stage from Chakrata-Deoban region, Uttarakhand (30.798624° N, 77.780368° E; altitude 2600 m) during the month of October, 2017 from wild. *Curcuma longa* L. rhizome was collected from Herbal garden, Lovely Professional University, Phagwara, Punjab. Both the plants were authenticated on the basis of morphological characters by Prof. R. C Gupta, taxonomist expert. The Voucher specimens of *Swertia chirata* (No. 01102017) and *Curcuma longa* (No. 11122017) were prepared and deposited in the Department of Botany, Lovely Professional University, Phagwara, Punjab, India.

### HPTLC instrumentation and chromatographic condition

HPTLC system (CAMAG) consists of 100 mL syringe (Hamilton, Bonaduz, Switzerland) and Linomat V automatic sample applicator (CAMAG, Muttenz, Switzerland). CAMAG glass twin trough chamber (20 cm × 10 cm × 4 cm) with stainless steel lid cover, CAMAG TLC Scanner III, CAMAG win Cats 3 integration software and UV cabinet with dual wavelength UV lamp (254 nm and 366 nm) was used for the HPTLC analysis. HPTLC analyses were performed on 20 × 10 cm pre-coated with silica gel 60 F254 (E-Merck) (0.2 mm thickness). HPTLC experiments were conducted at 25 ± 2 °C temperature and 40% relative humidity.

### Qualitative and quantitative determination of mangiferin, oleanolic acid, ursolic acid, gallic acid, quercetin and curcumin by HPTLC

#### Extraction and preparation of test samples for HPTLC

All the MC (Mahasudarshan churna) powdered samples (1.0 g) were extracted with microwave assisted extraction (MAE) method in 100 ml closed vessel units by using methanol solvent. Microwave extraction conditions were set at optimized conditions: microwave power (450 W), time (4 min), solid: solvent (20 mL).

Extracts were cooled, filtered through Whatman no. 1 filter paper and extracts were dried in rotary evaporator. Final volume of all the extracts was prepared with methanol (10 mg mL^− 1^), mixed well and then centrifuged the tubes for 5 min at 7500 rpm (4 °C) to get clear extracts. All the prepared extracts were kept in refrigerator at 4 °C for further phytochemical analysis. All the extraction procedures were completed in triplicates and their respective mean values were considered for yield.

#### Preparation of standard solution

The standards i.e. mangiferin, oleanolic acid, ursolic acid, gallic acid, curcumin and quercetin (10 mg each) were weighed accurately and standard solutions were prepared by adding 10 mL of methanol to produce 1 mg mL^− 1^ concentration of standards.

### HPTLC method development and validation for mangiferin, oleanolic acid, ursolic acid, gallic acid, quercetin and curcumin

The HPTLC plates were cleaned by predevelopment by using methanol and dried in oven at 105 °C before loading the test samples. Standards and samples were applied by means of applicator equipped with a Hamilton syringe of 100 μL capacity on HPTLC plates with the following settings: bands size, 5 mm; specific distance kept between the two bands, 14 mm; application rate, 150 nLs^− 1^; the distance from bottom of the plate 2 cm. Standard solutions of OA, UA, M, GA, Q and C were applied in triplicate on HPTLC plates using an automatic sample spotter (Linomat V) to get final concentration. The peak area was recorded and calibration curves were obtained by plotting peak area versus concentrations of the standards.

5 μL of each MC sample was loaded in triplicates on HPTLC plate. Optimized mobile phase for mangiferin (M), triterpenoids (OA, UA) and phenolics (GA, Q, C) were ethyl acetate: glacial acetic acid: formic acid: water (20: 2.2: 2.2: 5.2 v/v), n-hexane: ethyl acetate: acetone (16.4: 3.6: 0.2 v/v) and toluene: ethyl acetate: formic acid (13.5: 9: 0.6 v/v) respectively and plate were developed in twin-tough chamber (CAMAG) upto 7.5 cm. For OA and UA, HPTLC plate was pre-derivatized with 1% iodine solution in chloroform upto a distance of 3.5 cm, and was placed in dark for 10–15 min. The plates were dried with dryer to remove the excess of iodine after completion of the reaction. After development, HPTLC plate was post-derivatized with 5% (v/v) ethanolic sulphuric acid solution and after drying, heated in an oven at 110 °C for 2 min [15]. Quantitative evaluation of the plate was performed by keeping slit dimensions 4 × 0.33 mm, scanning speed, 20 mm s^− 1^ at a wavelength of *λ* = 254 nm for xanthanoid, *λ* = 520 nm for triterpenoid and *λ* = 254 nm for phenolics.

### HPLC analysis

Analysis was performed with HPLC-PDA detector System (Waters) equipped with an auto sampler, a dual low-pressure gradient system, C-18 column. HPLC conditions were optimized with isocratic elution and 1 mL/min flow rate. Working solutions of the samples were prepared at 100 ppm concentrations, and injection volume was set at 10 μL. Chromatographic separation was conducted at room temperature using Sunfire C-18 column (4.6 mm × 250 mm; 5 μm particle size). Before injection, column was equilibrated for 10 min.

For estimation of mangiferin, acetonitrile (A) was used as eluent A and water with 0.1% formic acid as eluent B using the following gradient program: 20–46% A (0–1.0 min); 46–46% A (1.0–2.5 min); 46–20% A (2.5–4.0 min) and 20–20% A (4.0–9.0 min). On the other hand, mobile phase for simultaneous determination of gallic acid and quercetin was consisted of water (A) and methanol (B). UV/Vis absorption spectra were recorded in the range of 200–600 nm. Identification of these compounds by chromatography was achieved by interpolation of multi-level standard curves made with authentic standards and retention time comparison. Quantitative analysis in UV/Vis based detection systems was accomplished using linear calibration curves generated with authentic standards of mangiferin, gallic acid and quercetin. Stock solutions of authentic standards (1 mg/mL) were prepared in pure methanol and made up to seven dilutions using methanol.

## Method validation

For the validation of the analytical method development, ICH guidelines were followed for precision, repeatability and accuracy.

### Instrument precision

Instrument precision was evaluated by analyzing the same spot of OA (600 ng/spot), UA (600 ng/spot), mangiferin (400 ng/spot), GA (600 ng/spot), Quercetin (400/spot) and curcumin (300 ng/spot) and expressed as relative standard deviation (%*RSD*).

### Precision

Precision was studied by analyzing six bands (*n* = 6) of both sample and standard solutions per plate on three plates (intra-day precision) and on three consecutive days (inter-days precision) at three different quantities (400, 600, 800 ng/spot for OA, UA and GA; 300, 400, 500 ng/spot for mangiferin and Quercetin; 200, 300, 400 ng/spot for curcumin).

The intra-day (repeatability) and inter-day (intermediate precision) precisions were performed by these three different concentration levels of standard and sample solutions and all results were expressed as mean ± *RSD (*%*)*.

### Limits of detection (LOD) and quantification (LOQ)

For the determination of LOD and LOQ, different concentrations of M, OA, UA, GA, Q and C were applied along with methanol as a blank and evaluated on the basis of signal-to-noise (S/N) ratio. The LOD was considered as 3:1 (SD/S) and LOQ as 10:1 (SD/S); S: slope of the calibration curve and SD: standard deviation of the Y-intercept of the regression line.

### Accuracy and recovery studies

The accuracy of the method was established by the measurement of the recovery of standards at three different concentration (50, 100 and 150%) of xanthanoid (mangiferin), triterpenoids (oleanolic acid, ursolic acid) and phenolics (gallic acid, quercetin and Curcumin) using the standard addition method.

The known volume of MC solution (each taken in four different funnel), mangiferin stock solution (1 mg mL^− 1^) equivalent to 0.0, 2.0, 4.0 and 6.0 μg were added individually to each of the separating funnels. Seven replicate analyses were performed on each extract and the amount of mangiferin recovered from the samples was determined for each level. The recovery analysis was repeated similarly for triterpenoids (oleanolic acid, ursolic acid) and phenolics (gallic acid, quercetin and Curcumin) with MC solutions.

### Specificity

Peak purity of the standard and test sample was determined for specificity of the method. The spot of each standard in the sample was confirmed by the *R*_*f*_ values and spectra of the separated bands with those of the standards at three different levels i.e. peak start, peak apex and peak end of the spot.

## Results

### Development of optimum mobile phase

**Fig. 1:**
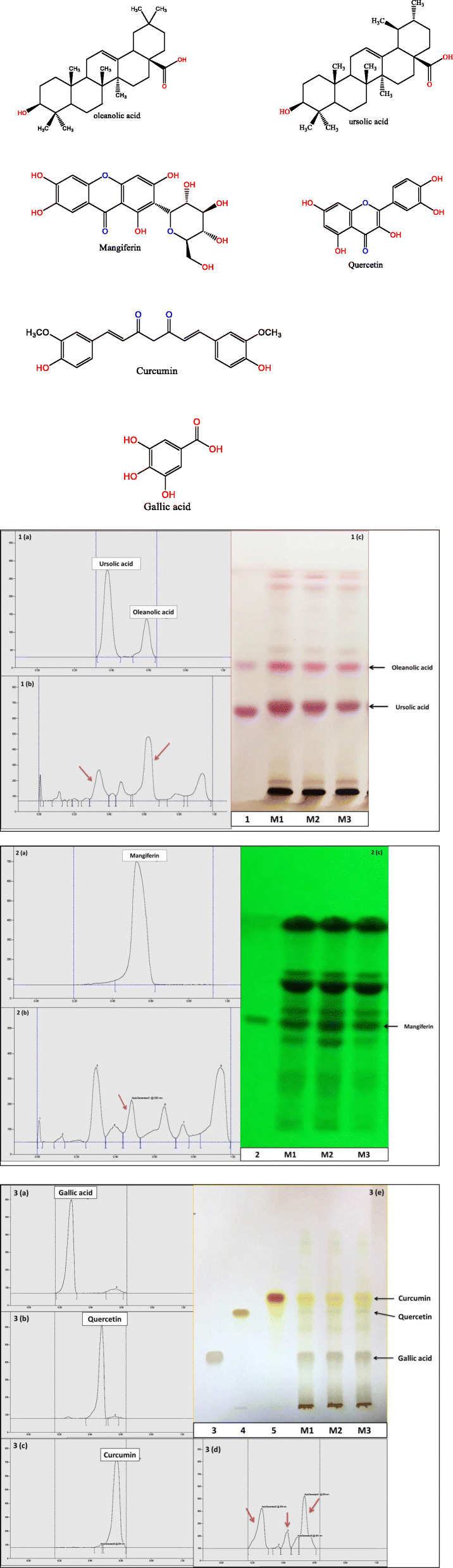
Densitogram obtained from Standard- ursolic and oleanolic acid (1 a) using n-hexane: ethyl acetate: acetone (16.4: 3.6: 0.2 v/v) mobile phase; formulation samples containing ursolic and oleanolic acid (1 b); Standard-mangiferin (2 a); formulation samples containing mangiferin (2 b) using ethyl acetate: glacial acetic acid: formic acid: water (20: 2.2: 2.2: 5.2 v/v) mobile phase; Standard- gallic acid (3 a); Standard- quercetin (3 b); Standard- curcumin (3 c) using toluene: ethyl acetate: formic acid (13.5: 9: 0.6 v/v) mobile phase; and formulation samples contains gallic acid, quercetin and curcumin (3 d). HPTLC fingerprinting (1c; 2c; 3e) showing track 1; 2; 3–5: STANDARD-oleanolic and ursolic acid; mangiferin; gallic acid; quercetin and curcumin respectively where tracks M1, M2 and M3 signifying marker compounds in three different commercial formulations (MC1, MC2 and MC3)

**Fig. 2:**
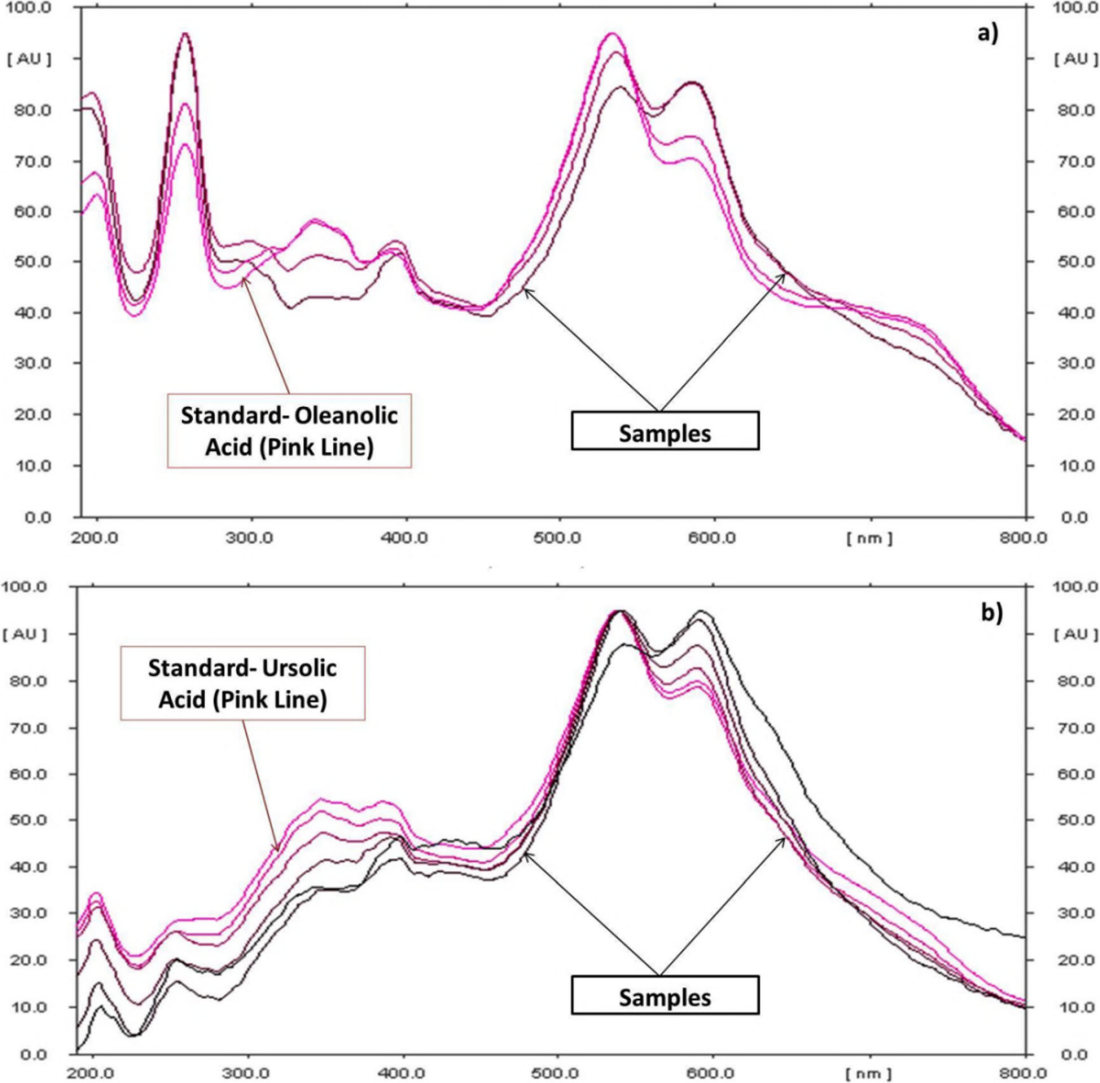
Spectrum of oleanolic acid a) and ursolic acid b) at 520 nm superimposed with the formulation samples

**Fig. 3:**
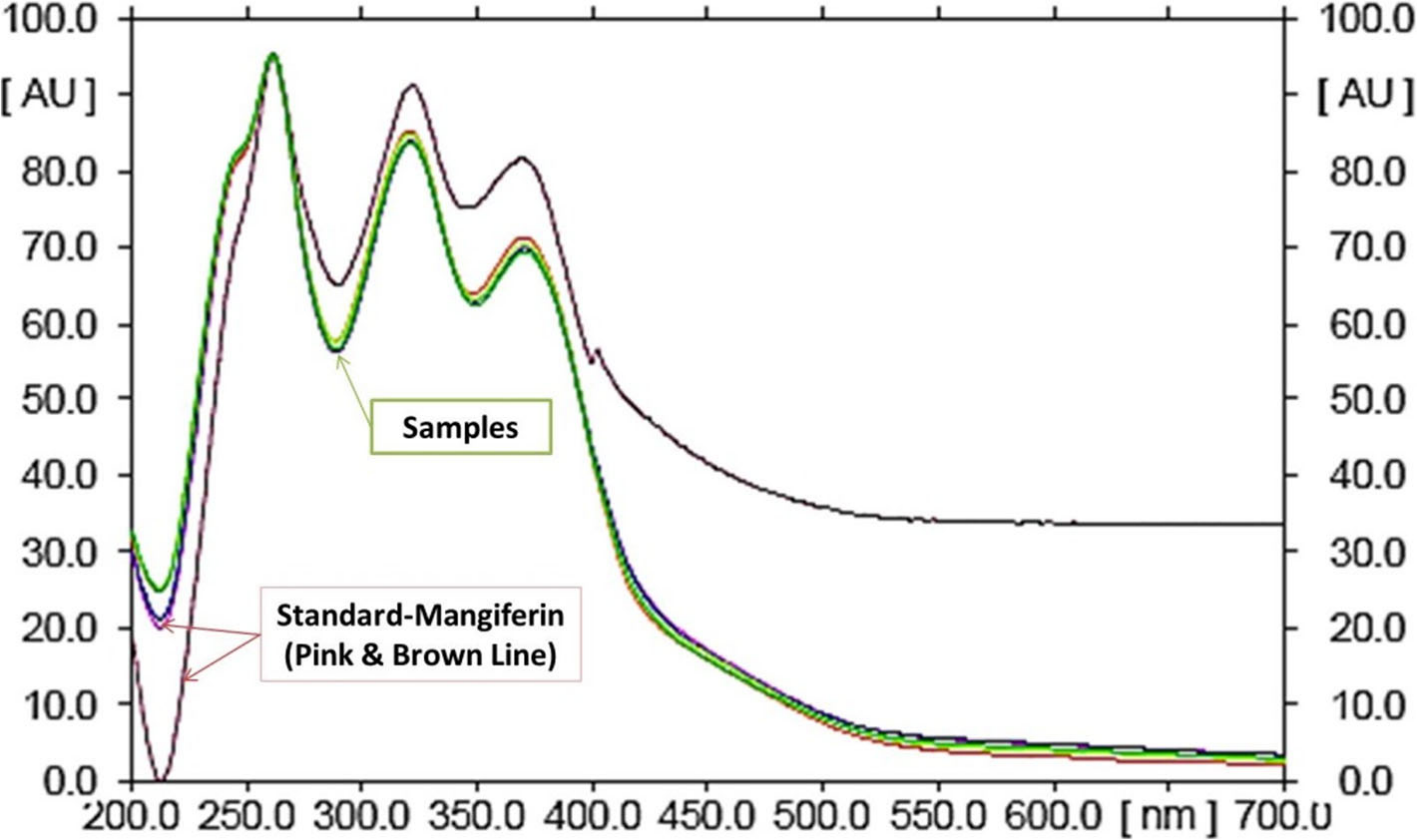
Spectrum of mangiferin at 254 nm superimposed with the formulation samples

**Fig. 4:**
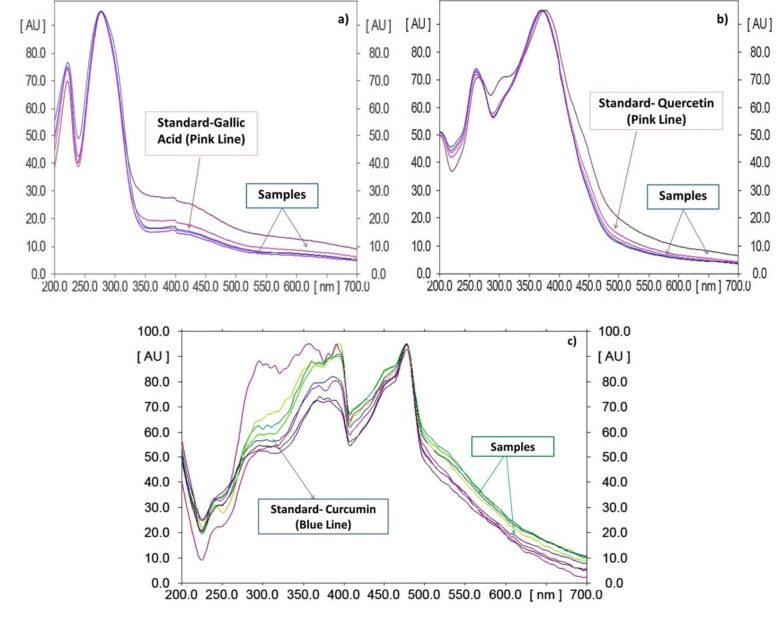
Spectrum of gallic acid a), quercetin b) and curcumin c) at 366 nm superimposed with the formulation samples

In this study, quantitative estimation of specific bioactive compounds viz. M, OA, UA, GA, CA and curcumin was conducted in the commercial polyherbal formulations using HPTLC technique. In order to optimize the best solvent system for good resolution and separation of chosen bioactive compounds, various trials were carried out. Well resolved spot with significant R_f_ for determination of xanthonoid (M), triterpenoids (OA, UA) and phenolics (GA, Q and C) in formulations was developed. M, OA, UA, GA and Q, the common phyto-constituents in *Swertia chirata* and curcumin, the major bioactive marker compound of *Curcuma longa,* were well represented in the chromatograms (Fig. 1 a, b, c). For optimization of method, different mobile phase compositions were employed to achieve good separation and resolution of these bioactive compounds. Of different solvent systems used for the detection of M, OA, UA, GA, Q and C, the solvent system containing ethyl acetate: glacial acetic acid: formic acid: water (20: 2.2: 2.2: 5.2 v/v/v/v), n-hexane: ethyl acetate: acetone (16.4: 3.6: 0.2 v/v) and toluene: ethyl acetate: formic acid (13.5: 9: 0.6 v/v) resulted in good resolution of the xanthanoid, triterpenoids and phenolics in the presence of other compounds in the formulation. The R_f_ obtained for oleanolic acid is 0.59 and ursolic acid is 0.38. The R_f_ of mangiferin is 0.53. R_f_ obtained for gallic acid, quercetin and curcumin is 0.28, 0.48 and 0.56, respectively. The R_f_ value of the triterpenoids (OA, UA), xanthonoid (mangiferin), and phenolics (GA, CA and curcumin) in different solvents system with reference standard and polyherbal formulations (MC) were found comparable under 520 nm (Fig. 2), 254 nm (Fig. 3), and 366 nm (Fig. 4) respectively. The TLC chamber was pre-saturated with the mobile phase for 5 min at room temperature to get well-defined band. The three-dimensional HPTLC overlay of triterpenoids (OA, UA), xanthonoid (M), and phenolics (GA, Q and C) are shown in Figs. 2, 3 and 4.

In addition, HPLC-PDA analysis was used to confirm the HPTLC results (Additional file 1: Fig. S1 and S2).

### Calibration curves of Mangiferin, Oleanolic acid, Ursolic acid, gallic acid, quercetin and Curcumin and their analysis in formulations

Calibration curve was found to be linear at 200–800 ng/band for mangiferin, 200–3000 ng/band for triterpenoids (OA and UA), 500–2000 ng/band for gallic acid, 300–1500 ng/band for quercetin and 100–1000 ng/band for curcumin. The results are tabulated in Table 1. The precision of xanthonoid (M), triterpenoids (OA, UA) and phenolics (GA, Q and C) are shown in Table 2. The intra-day and inter-day precision results are found to be precise as shown in Table 2.

**Table 1:** Method validation parameters for the quantification of Mangiferin, Oleanolic acid, Ursolic acid, Gallic acid, Curcumin and Quercetin

Sr.no.	Parameters	Mangiferin (M)	Ursolic acid (UA)	Oleanolic acid (OA)	Gallic acid (GA)	Quercetin(Q)	Curcumin(C)
1	Linearity range (ng/spot; *n* = 12^a^	200–800	200–3000	200–3000	500–2000	300–1500	100–1000
2	Correlation coefficient (*r*^*2*^)	0.987	0.991	0.997	0.988	0.992	0.99
3	Regression equation	Y = − 608.4 + 9.033X	Y = − 126.87.0 + 11.05X	Y = − 1111.13 + 10.0914X	Y = 1939 + 7.19X	Y = −200 + 1.74X	Y = 137 + 9.66X
4	Calculated SD value (CATS software)	6.72	2.59	3.26	5.33	2.12	2.43
5	^b^Limit of detection (LOD) (ng) [3 x SD/S]	25	30	27	40	45	30
6	^b^Limit of quantitation (LOQ) (ng) [10 x SD/S]	75	90	81	125	140	90
7	R_f_	0.53	0.38	0.59	0.28	0.48	0.56
8	λmax	254 nm	520 nm		366 nm		
9	Mobile phase	Ethyl acetate: glacial acetic acid: formic acid: water (20: 2.2: 2.2: 5.2 v/v)	n-hexane: ethyl acetate: acetone (16.4: 3.6: 0.2 v/v)		Toluene: ethyl acetate: formic acid (13.5: 9: 0.6 v/v)		

^a^ Four concentration levels in triplicate; ^b^SD is the standard deviation of the blank response and S is the slope of the calibration plot

**Table 2:** Analytical characteristics of the method validation

Sr. no.	Parameters	Mangiferin (M)	Ursolic acid (UA)	Oleanolic acid (OA)	Gallic acid (GA)	Quercetin (Q)	Curcumin (C)
	**Precision and accuracy**						
1	Intra-day RSD (%), *n* = 5	0.437	0.357	0.487	0.67	0.56	0.45
2	Inter-day RSD (%),*n* = 5 (day-1/day-2/day-3)	0.437/0.495/0.512	0.353/0.425/0.532	0.484/0.498/0.514	0.67/0.68/0.71	0.56/0.59/0.63	0.45/0.48/0.49
	**Recovery**						
3	Amount of standard in MC samples (μg mg^−1^) containing highest bioactive compounds	44.6	8.1	19.2	20.1	10.3	6.1
4	Amount of standards added in MC sample (μg mg^− 1^)	20/40/60	4/8/12	10/20/30	10/20/30	5/10/15	3/6/9
5	Amount of standard found (μg mg^−1^)	64.36/84.4/104.26	12.1/16.2/20.11	29.21/39.19/49.12	30.12/40.13/50.03	15.29/20.28/25.33	9.1/12.21/15.09
6	Recovery (%)	99.62/99.76/99.67 (99.81)	100/100.62/100.04	100.03/99.97/99.83	100.06/100.07/99.86	99.93/100.89/99.9/100.11	100/100.9/99.93

### Quantification of bioactive compounds in commercial Mahasudarshan Churna formulations by HPTLC and HPLC

HPTLC method was used for qualitative and quantitative estimation of six major bioactive compounds; viz. oleanolic acid, ursolic acid, mangiferin, gallic acid, quercetin and curcumin in all the test samples of MC. The chromatograms and HPTLC fingerprints of the all standard compounds along with crude extract of MC samples are presented in Fig. 1. MC formulations (MC1, MC2 and MC3) were found to contain oleanolic acid (1.54–1.78%), mangiferin (1.38–1.52%) and gallic acid (1.01–1.15%), followed by ursolic acid (0.79–0.98%), curcumin (0.45–0.67%) and quercetin (0.22–0.34%) content with HPTLC analytical method.

In addition, HPLC-PDA analysis was used to confirm the HPTLC results. In present study, quantification of only three compounds; viz. mangiferin, gallic acid and quercetin was performed using HPLC method. In this method, MC formulations (MC1, MC2 and MC3) were found to contain mangiferin (1.36–1.54%); gallic acid (1.06–1.12%) and quercetin (0.20–0.36%) content.

### Limit of detection and quantification

The LOD and LOQ of xanthonoid (M), triterpenoids (OA, UA) and phenolics (GA, Q and C) are shown in Table 1 which indicates the adequate sensitivity of the method.

### Accuracy and recovery studies

Accuracy was investigated by the application of 50, 100 and 150% of xanthonoid (M), triterpenoids (OA, UA) and phenolics (GA, Q and C) in the Mahasudarshan churna formulations extract. Average recovery for xanthonoid (mangiferin), triterpenoids (oleanolic acid and ursolic acid) and phenolics (gallic acid, quercetin and curcumin) for the marketed formulation of Mahasudarshan churna are depicted in Table 2.

### Specificity

The peak purity was calculated as per regression (*r*^2^). The values for mangiferin, OA, UA, GA, quercetin and curcumin are presented in Table 2. Chromatographic specificity was investigated by comparing the *R*_*f*_ value of standard compounds and test samples and it was found to be identical. The specificity of the method is checked and no impurities or degradation products were found in samples and the peaks of standard drug solutions.

## Discussion

Herbal medicines (HMs) including Chinese medicine (CM) and other folk medicines are getting more and more popular around the world nowadays, in order to improve the health condition of human beings. The use of medicinal plants as therapeutics is the oldest prevalent system to deal with illness, but still there is a lack of proper standardizing techniques for the assessment of their quality, quantity and efficiency [19]. According to WHO guiding principle, chromatographic procedures are most effective for the standardization and quantification of value-added bio-active compounds from herbal/polyherbal formulations [3].

Mahasudarshan Churna (MC) is a polyherbal Ayurveda medicine, which is known for antimalarial, antioxidant, antipyretic, antiviral and anti-diabetic properties [8]. *Swertia chirata* is known as principal component of MC; however, framework for the quality control and standardization protocols of this formulation is still unclear. Xanthonoids (mangiferin), secoiridoids (swertiamarin and amarogentin) and triterpenoids (oleanolic acid and ursolic acid) are bio-markers compounds of *Swertia* genus [12, 13]; therefore, this study was designed for the qualitative and quantitative analysis of major bio-active compounds in MC.

In present study, a validated Densitometric- HPTLC (high performance thin layer chromatography) method has been developed for the determination of major bio-markers (xanthonoid-mangiferin, triterpenoids- oleanolic acid and ursolic acid, phenolic compounds-gallic acid, quercetin and curcumin) in Mahasudarshan Churna (MC) formulation. The proposed method is simple, precise, specific, accurate, less time consuming and cost effective. Statistical analysis proved that the method is evitable for the analysis of xanthonoid, pentacyclic triterpenoids and phenolic compounds. The developed HPTLC method will help the manufacturer for standardization and routine quality control of raw materials and herbal products containing *Swertia chirata* and rhizome of *Curcuma longa* as an ingredient. Polyherbal formulations consist of multifarious mixture of phytoconstitutes wherein no single component is responsible for overall efficacy [9]. This causes a challenge in quality control standards for raw materials and standardization of herbal formulations [20]. Selecting one or more markers is a common practice in natural product analysis for purposes of identification and quality assessment [21].

In present study, three major bioactive compounds (mangiferin, oleanolic acid and ursolic acid) of *S. chirata*; one bio-marker compound (curcumin) of *Curcuma longa* and significant phenolic compounds viz. gallic acid and quercetin were present in significant amount in all the commercial test samples of MC. Analysis of bio-active compounds in the present study was performed using HPTLC methods and later HPTLC results were compared with HPLC. These two methods give comparable results and there was no statistically significant difference between the mean values for all extracts. Consequently, both HPTLC densitometry and the HPLC method could be used for the qualitative and quantitative estimation of these bio-active compounds in Mahasudarshan churna (MC) extracts. However, relatively HPTLC was found to be simple, fast and cost-effective method.

Our previous studies reported the presence of substantial amount of mangiferin and triterpenoids in *Swertia chirata* plant samples [12–14]. Kaur et al. [12, 13] quantified 3.87% ursolic acid, 3.27% oleanolic acid and 4.31% in aqueous ethanolic extracts of wild *S. chirata* collected from Western Himalayas, India*.* Pandey et al. [14] also reported 4.37% mangiferin yield in methanolic extracts of *S. chirata* wild samples collected from Eastern Himalayas, India. Moreover, wild sample of methanolic extract of *Curcuma longa* yielded 5.38% content. *S. chirata* is considered as the most prolific source of important bioactive compounds [11]. Significant amount of ursolic acid, oleanolic acid and mangiferin compounds has been quantified in other *Swertia* species viz. *S. angustifolia, S. alata, S. paniculata, S. minor, S. densifolia, S. lawii, S. corymbosa* etc. [22, 23]. *S. chirata* and other *Swertia* species have been widely used in the Ayurvedic, Unani and Chinese herbal formulations for treating fever, malaria, epilepsy, ulcer, asthma, liver disorders, hepatitis, diabetes etc. as tinctures, infusions etc. [24].

Presence of significant marker compounds in MC formulations is in accordance with our previous reports of *Swertia chirata* herbs. Therefore these bio-marker compounds are authentic and can be efficiently used for the standardization of this polyherbal formulation.

## Conclusion

A new HPTLC method has been developed and validated for the quantification of xanthonoid (mangiferin), triterpenoids (oleanolic acid and ursolic acid) and phenolics (gallic acid, quercetin and curcumin) in marketed formulations of mahasudarshan churna containing *Swertia chirata* as a major ingredient. From the above mentioned results, it can be concluded that HPTLC technique is low cost, fast, precise, and accurate which can be successfully employed for the quantification of xanthonoid (mangiferin), triterpenoids (OA, UA) and phenolics (GA, quercetin and curcumin). Furthermore, the develop HPTLC method can be conveniently employed for routine quality control analysis of all the six marker compounds for marketed formulations in Ayurvedic/Herbal industry.

## Supplementary information

**Additional file 1.**

## Data Availability

The datasets used and/or analysed during the current study available from the corresponding author on reasonable request.

## Abbreviations

MC: Mahasudarshan Churna
OA: Oleanolic acid
UA: Ursolic acid
GA: Gallic acid
C: Curcumin
Q: Quercetin
M: Mangiferin
HPTLC: High performance thin layer chromatography.
