# Supplementary material for: Validation and quantification of major biomarkers in ‘Mahasudarshan Churna’- an ayurvedic polyherbal formulation through high-performance thin-layer chromatography
Source: BMC Complement Med Ther. 2020 Jun 11;20:184. doi: 10.1186/s12906-020-02970-z (PMC7291524; doi:10.1186/s12906-020-02970-z)
Supplement: Supplementary file 1 — Additional file 1. [file 12906_2020_2970_MOESM1_ESM.docx]

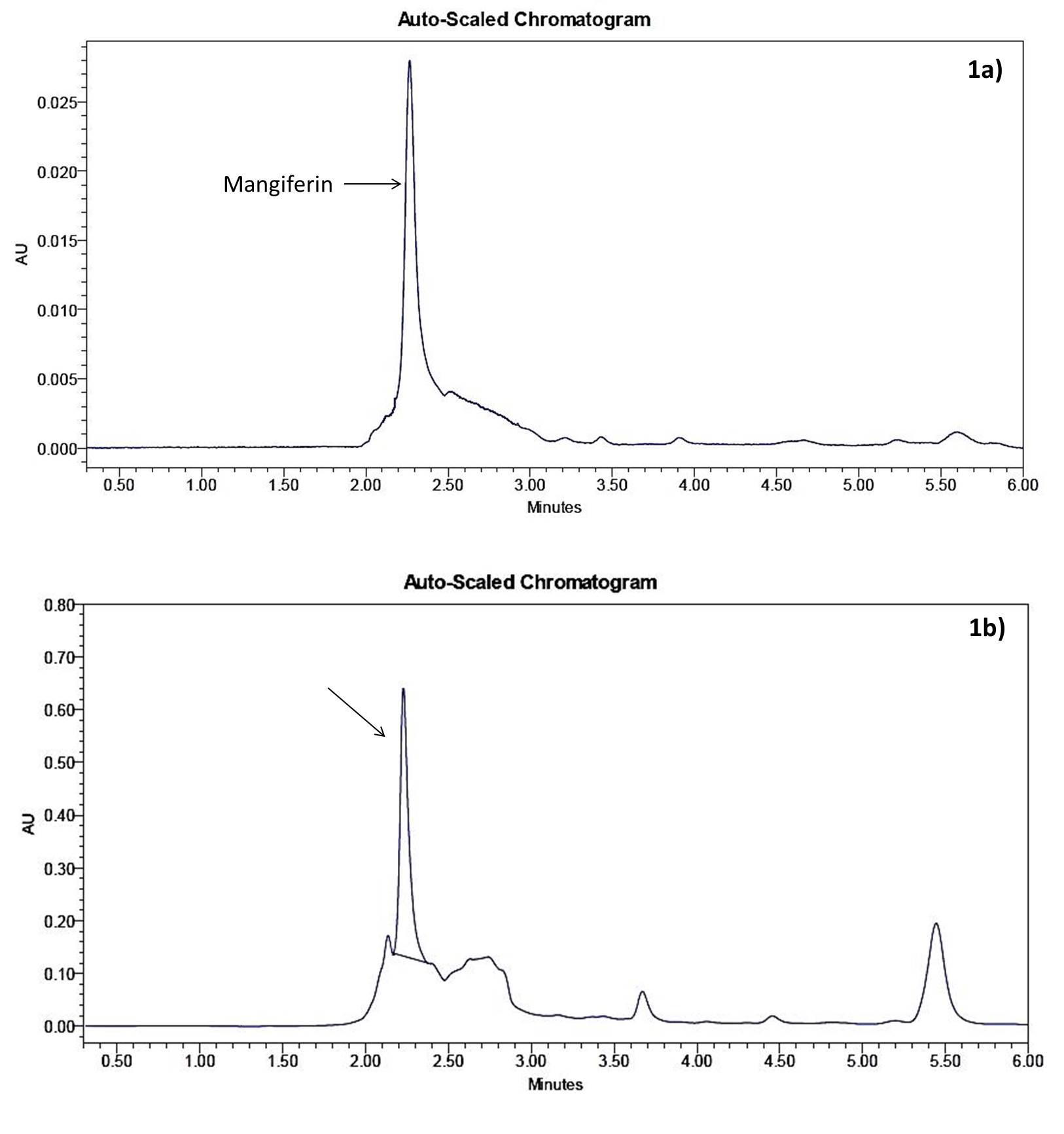


Figure S1 HPLC chromatogram of a mangiferin: standard compound (1a) whereas (1b) represents HPLC chromatograms obtained from Mahasudarshan Churna (MC) sample.


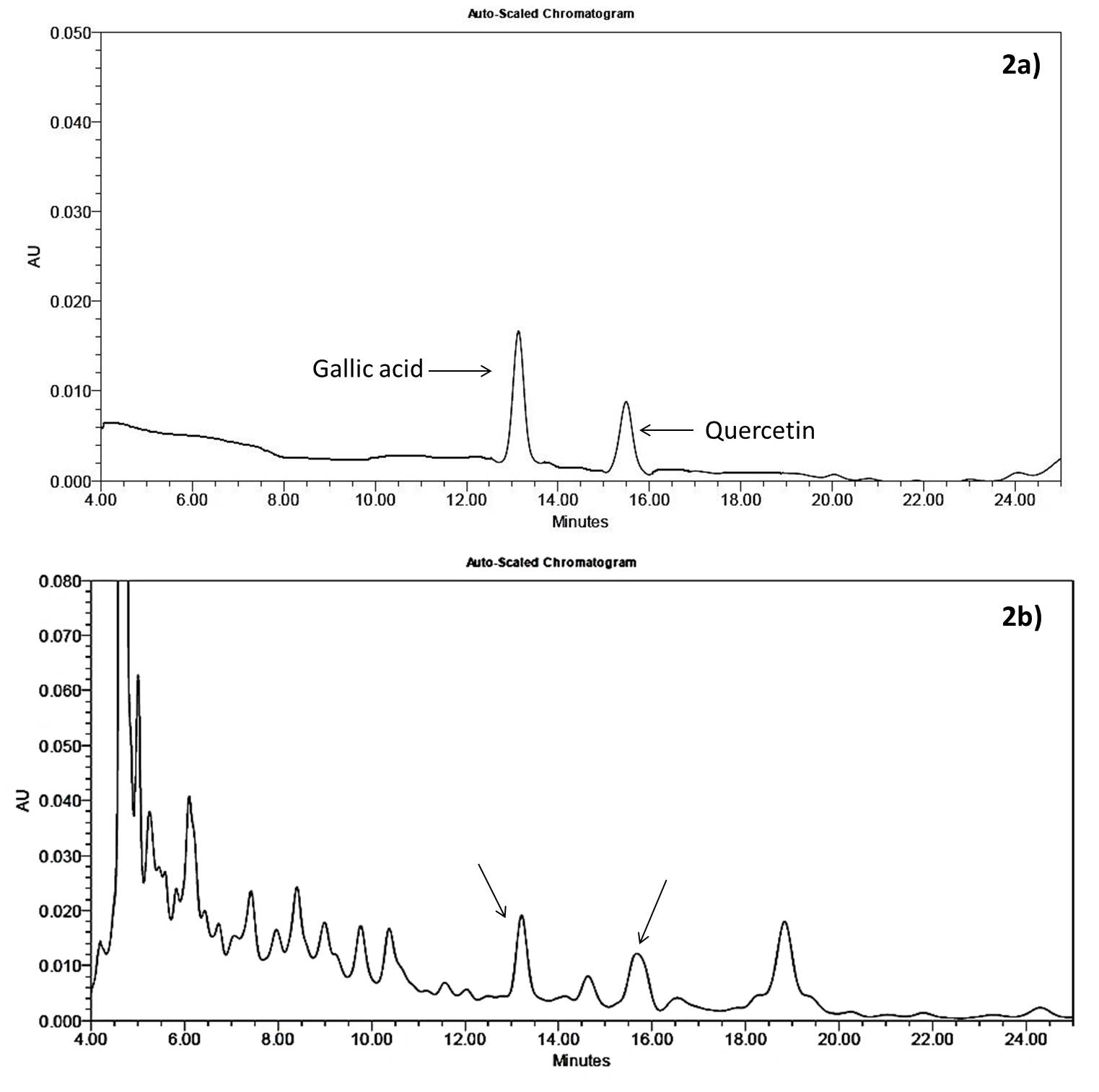


Figure S2 HPLC chromatogram of a gallic acid and quercetin: standard compound (2a) whereas (2b) represents HPLC chromatogram obtained from Mahasudarshan Churna (MC) sample.
